# Neurological Manifestations of COVID-19 Within the Intensive Care Unit During a Military Deployment for the Early Pandemic Surge in New York City

**DOI:** 10.7759/cureus.13858

**Published:** 2021-03-12

**Authors:** Richard P Menger, Ian L Valerio

**Affiliations:** 1 Neurosurgery and Political Science, University of South Alabama, Mobile, USA; 2 Plastic and Reconstructive Surgery, Massachusetts General Hospital, Boston, USA

**Keywords:** covid-19 outbreak, new york city, us navy, military physician, stroke, covid coagulopathy, stroke and covid-19, medical intensive care unit (micu), deployment medicine, operational medicine

## Abstract

Coronavirus disease 2019 (COVID-19) resulted in a worldwide pandemic that at the time of this writing has caused over 400,000 deaths within the United States. During the pandemic surge in New York City, NY, a number of military Medical Corps (MC) and Nurse Corps (NC) providers were mobilized in direct support of critical care capabilities through expansion intensive care units. In the course of the deployment, high rates of neurological-related manifestations associated with COVID-19 infection were directly observed by our military provider teams which will be described and supporting literature highlighted.

This is organic information absorbed in real time during the early stages of the pandemic in New York City.

The neurological manifestations of COVID-19 varied in presentation and severity. Cerebral vascular injuries documented included strokes, iatrogenic intraparenchymal hemorrhage, hypoxia-related changes and sequelae, as well as acquired diseases secondary to delayed treatment of other primary neurologic disease states. Hypercoagulable and inflammatory markers (d-dimer, C-reactive protein, etc) were commonly elevated, and anticoagulation became a key factor in disease treatment and to help mitigate the downstream neurologic sequelae associated with this disease.

Here we present these initial findings to lay the groundwork for more robust clinical studies moving forward.

## Introduction

Triservice military medical teams were rapidly mobilized to provide aid and services during the coronavirus disease 2019 (COVID-19) pandemic surge in New York City, NY, of 2020. These military medical assets consisted of physicians, nurses, and associated healthcare support staff who were assigned to platforms including the Navy Medicine Support Team (NMST), the Army Urban Augmentation Medical Task Forces (UAMTFs), and Air Force Reservists which were integrated into various New York City (NYC) civilian hospitals. At the time of drafting of this manuscript, the city of New York had documented over 207,693 confirmed cases and 17,127 COVID-19-associated fatalities [1]. At this same particular point in time, deaths in NYC surpassed all other cities in the world and totaled more than all but six countries [2]. The state of New York had logged over 378,799 cases with 24,299 COVID-19-associated deaths during this same observed time period, highlighting the severity of the outbreak. 

In April 2020, nearly 1600 U.S. Navy Reservists were mobilized in support of COVID-19 alone [3]. Specifically, 221 physicians and nurses were mobilized as Individual Augmentees (IAs) to serve on the NMST mission and were embedded into the public city-funded NYC Health and Hospital (NYHH) system. Many were re-deployed into deeply versatile positions stepping out of their traditionally defined specialties and training roles in expansion of critical care services through manning and/or setting up surge intensive care units (ICUs) based on the needs of the respective hospitals. This particular Navy mission model was a new and expansive role for military medicine and military neurosurgery [4].

Given the early experiences of our team, a number of neurological and neurosurgical diseases, most predominantly stroke and hemorrhage, presented during the management of COVID-19 ICUs. Within a large public city hospital in NYC, identification of these neurological manifestations and establishment of treatment protocols was critical in reducing short- and long-term morbidity associated with neurologic injury associated with COVID-19 infection. These manifestations will be outlined in the following sections with suggested treatment considerations with supporting literature reported. 

## Case presentation

Cerebrovascular accidents and strokes

Continuing evidence introduces the pro-thrombotic and inciting microvascular injury and nature that COVID-19 infection may manifest. Mao et al. documented some of the earliest studied neurologic presentations and observations of hospitalized COVID-19 patients treated in Wuhan, China. Across 214 patients, 45.5% had a neurologic presentation with COVID-19 infection which included acute cerebrovascular events, impaired consciousness, and/or neuromuscular injuries. Severity of disease was defined primarily by respiratory symptomology based on community-acquired pneumonia standards. Of those patients suffering from severe COVID-19 infection sequelae, 5.7% of patients had an acute cerebrovascular disease as confirmed via chart review and/or supported by imaging analysis. Other associated symptoms consisted of loss of taste or ageusia in 5.6% and loss of smell or anosmia in 5.1% of cases, respectively, including both severe and less acuity cases having confirmed COVID-19 infection [5].

These observed data have been reproduced in various studies within the United States. For example, Oxley et al. presented case series level data explicitly showing stroke in patients less than the age of 50. These neurologic injuries were determined to be large vessel strokes in patients ranging in age from 33-49 years old. A 33-year-old patient illustrated in this publication specifically delayed seeking care within the mechanical intervention window secondary to concerns of COVID-19 risk if she were to present to a medical center for evaluation and/or treatment. Neurological treatment of cases reported in this series included supportive care measures, basic and advanced medical therapies, certain endovascular procedures, and in one case surgical care via need for a hemicraniectomy [6]. 

In another large series reported by Zhou et al., 191 cases from both Jinyintan Hospital and Wuhan Pulmonary Hospital were evaluated for causes of mortality with a direct correlation to inflammatory status identified. Multivariable regression analysis showed increased odds of in-hospital death associated with older age (odds ratio 1·10, 95% CI 1·03-1·17, per year increase; p=0·0043), higher Sequential Organ Failure Assessment (SOFA) score (5·65, 2·61-12·23; p=0.0033), and d-dimer measurements greater than 1 µg/mL (18·42, 2·64-128·55; p=0·0033) on admission. Nearly 48% of cases had a medical comorbidity [7].

**Figure 1:**
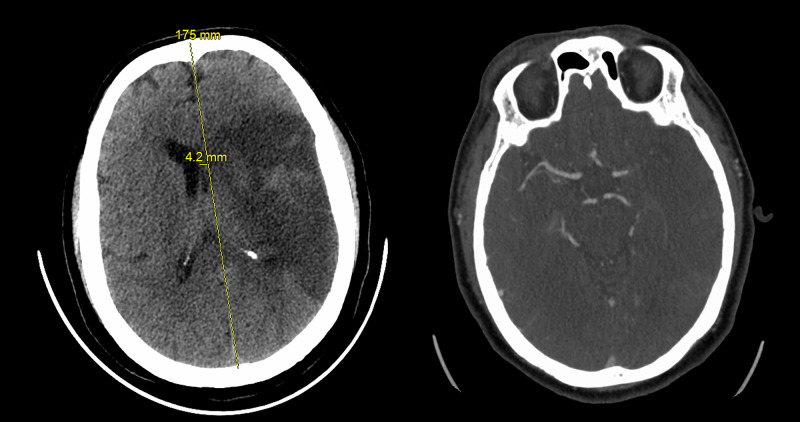
39-year-old male with left-sided first segment middle cerebral artery occlusion

In support of the aforementioned studies, the following stroke case was treated within our ICU. A 39-year-old male with no significant past medical history presented to the emergency room suffering from hemiplegia following a night of excessive alcohol intake (see radiographic images of Figure 1). This particular patient lived in a highly burdened community where widespread COVID-19 infection was noted, and he directly had several sick contacts in his home setting. Of note, his COVID-19 polymerase chain reaction (PCR) testing was inconclusive, which was not an uncommon finding as the sensitivity of PCR testing hovered around 71% in the early COVID-19 testing kits [8]. Radiographic imaging showed a right-sided proximal M1 segment occlusion of the middle cerebral artery. Given the delayed presentation to the hospital, the patient was not a candidate for mechanical thrombectomy as he had missed the interventional window. He had a significant thrombotic burden as supported by bilateral lower extremity thrombosis along with an accompanying right upper extremity superficial thrombosis on imaging studies. This presenting situation created a dynamic clinical management challenge as the patient was still within the surgical intervention window for a decompressive craniectomy, however, he also had an associated exceptionally high thrombotic burden and significantly elevated surgical risk. The patient underwent urgent placement of an inferior vena cava filter which proved technically challenging given the mechanical venous blockage that develops in patients having this level of thrombotic disease. The decision to transition to full dose anti-coagulation was done at two weeks as recommended by the multidisciplinary team with specific input from our neurology colleagues. 

Intraparenchymal hematoma (IPH)

Anecdotally, two-thirds to three-fourths (66-75%) of ICU patients with COVID-19 infection are on therapeutic doses of anti-coagulation via either oral, transdermal, and/or intravenous routes. Patients on therapeutic anticoagulation possess existing concern for transformation of spontaneous intracerebral hemorrhages (IPHs). In fact, this scenario represents the second most common subtype of stroke seen, and fatality in these cases can reach over 60% within one year [9,10]. Spontaneous IPHs can occur more often in patients having supratherapeutic anticoagulation levels although they have been documented in patients having normal therapeutic ranges as well. Indeed, a case of a spontaneous IPH fatality was appreciated during our deployment with a rapid Glascow Coma Scale decline in a patient treated for deep vein thrombus. 

In recent reports, a role for extracorporeal membrane oxygenation therapy for certain severe COVID-19-infected patients may exist, however, a known complication of this therapy includes IPH [11]. Finally, COVID-19 infection itself has also been postulated in relationship with IPH secondary to its use of the angiotensin-converting enzyme (ACE) II receptor for cell entry [12]. This hypothesis is an area of interest for further investigation. 

COVID-19-associated hypoxia

From an incidence standpoint, the most common neurological presentation associated with COVID-19 infection is brain hypoxia secondary to the pulmonary restraints and respiratory distress this disease commonly manifests. For example, a 65-year-old male with a medical history significant for hypertension, chronic obstructive pulmonary disorder, and asthma presented six days prior to hospitalization with symptoms of fatigue, body aches, anorexia, fevers, cough, and progressive dyspnea or shortness of breath. Over the next four days his symptoms progressed, which necessitated his presentation to his primary care provider with worsening of his baseline wheezing and increased exertional dyspnea and shortness of breath. He was noted to have a positive COVID-19 PCR test and was subsequently started on azithromycin and prednisone. On the day of his hospital admission, he was found by his wife with cyanotic hands and frothing as well as sputum coming from his nasal and oral airways. Emergency medical service (EMS) was initiated, and upon EMS arrival the patient’s initial oxygen saturation was noted to be in the 30s. The patient was transferred to the emergency room where additional laboratory and radiologic findings showing a white blood cell count of 23,000, a C-reactive protein level of 116, a d-dimer level of 512, and a chest x-ray showing bilateral infiltrates. Care was rapidly escalated to inpatient ICU admission and initiation of intravenous antibiotic therapy of vancomycin, piperacillin/tazobactam, and metronidazole for concerns of superimposed pneumonia on top of COVID-19 infection was implemented.

On hospital day #1, the patient was acutely found to be bradycardic to the 50s and hypoxic to an oxygen saturation of the 40s. Noted to be in asystole, advanced cardiac life support (ACLS) was initiated with return of spontaneous circulation (ROSC) in 10 minutes. The patient experienced high plateau pressures requiring switch from volume control to pressure control ventilatory management. As was apparent in a number of individuals requiring high positive pressures, the patient unfortunately suffered a pneumothorax necessitating chest tube placement. He required increasing vasopressor support, neuromuscular paralytics, steroids, and was placed in a prone position for persistent hypoxia and an arterial oxygen partial pressure/fractional inspired oxygen (PaO2/FiO2) <100. The steroids resulted in a transient diabetic ketoacidosis and GI-related bleed treated with pantoprazole IV drip. His accompanying myoclonic jerks were treated with lorazepam. After his three-day ICU course of care, he had persistent hypoxia with oxygen saturation in the 70s despite maximized ventilatory settings. Unfortunately, the patient’s neurologic status deteriorated with a CT scan of the head showing patchy hypodensities along all watershed areas concerning for diffuse hypoxia, and the patient eventually expired due to his hemodynamic instability coupled with neurologic injury.

Hospital-acquired COVID-19 transmission

Concern for hospital-acquired COVID-19 infection has been seen and validated [13]. This included patients presenting at our Level 1 Trauma Centers in NYC and elsewhere. While extreme concern and care were taken to separate patients with and without COVID-19 as much as possible, hospital-acquired COVID infections were seen. This situation was openly challenging during the intense surge of disease presentation. Specific COVID-19 overflow units were created. Indeed, in England, up to 20% of patients with COVID-19 were considered to be hospital-acquired cases [14]. This will become a more pressing issue moving forward as hospitals and health systems understand the reality related to maintaining typical healthcare in the backdrop of an ongoing pandemic. Of note, no significant neurologic symptoms or issues presented in those medical and nurse corps officers deployed in support of NMST. Strict and absolute adherence to personal protective equipment protocols was mandatory. This included N-95 masks, gowns, gloves, hair coverage, and shoe coverage.

Delayed care scenarios

Significant concern in both the lay and public press surrounds the concern for patients delaying necessary care out of fear of acquiring COVID-19 infection. As reported within the Oxley et al. series, one particular patient with a large vessel stroke missed her stroke intervention window due to such concerns [6]. In a report out of Italy, visits to pediatric emergency departments decreased 73-88% in comparison to the same time periods in 2019 and 2018, respectively, which was associated with some parents noting not wishing to bring their child to hospitals given high COVID-19 exposure risks [15]. Similar concerns have been highlighted and reproduced in the oncologic care population as well [16]. This was probably more ubiquitous in the vulnerable patient population served by the New York City Health and Hospitals.

**Figure 2:**
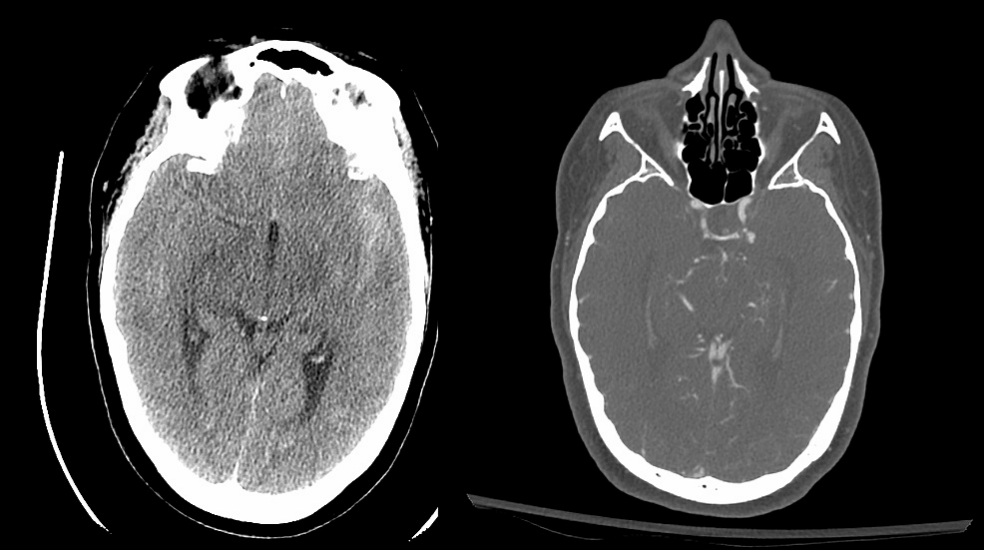
47-year-old female with ruptured L posterior communicating artery aneurysm

Our medical care team witnessed such cases during our deployment. Specifically, a 47-year-old female presented to our ICU as a transferred from the ER with “the worst headache of her life”. She delayed going to a hospital for six days since her headache onset despite having intermittent hemiplegia and confusion due to concerns of COVID-19 exposure risk. She was noted to have a Hunt and Hess Grade 3, Fischer grade 1 subarachnoid hemorrhage with hydrocephalus illustrated by the prominence of the temporal horns bilaterally on imaging. CT angiography and subsequent formal angiography illustrated a ruptured posterior communicating artery aneurysm that was coiled via endovascular intervention. This is appreciated in Figure 2.

## Discussion

Lessons learned and next steps…

As highlighted through the supporting literature and case-based examples, the mobilization of our military medical teams into areas of COVID-19 pandemic outbreaks has presented interesting medical treatment challenges and opportunities to reflect and apply lessons learned to future pandemic responses. Naturally, solutions to the proposed challenges of COVID-19 will develop as more is understood regarding the virus and its disease process. However, certain neurological manifestations associated with COVID-19 infection are important to identify as appropriate treatment will aid in lessening neurological-associated morbidity and mortality. Neurologic sequelae related to hypoxia require rapid intervention and support in improving the underlying respiratory status and condition. The cellular and microvascular damage caused by the virus has obvious deleterious effects directly on the pulmonary system which directly contributes to the hypoxic state commonly seen in these patients. Prone positioning has been shown to improve survival as well as permit the posterior and inferior lung spaces to be less restricted and thus improve oxygen exchange for many patients suffering from COVID-19 infection. Additionally, certain therapeutic medications have beneficial impact on improving respiratory function. Recent data from the U.K. and supported through our ICU early experiences illustrated that steroids such as dexamethasone reduced deaths by one-third in ventilated patients (rate ratio 0.65 [95% confidence interval 0.48 to 0.88]; p=0.0003) and by one-fifth in other patients receiving oxygen only (0.80 [0.67 to 0.96]; p=0.0021) [17]. With a treatment of 6mg orally of dexamethasone daily, one death would be prevented by treatment for around eight ventilated patients [17]. More studies will continue to progress our understanding around managing the evolving respiratory component of this disease, which will aid in reducing end-organ damage secondary to hypoxia.

Attention and care in neurologic examinations can provide early warning signs in the setting of intraparenchymal hematoma and stroke. Early management changes that could improve outcomes, e.g. stopping anticoagulation in patients with IPH or in providing mechanical thrombectomy and pharmacologic anticoagulation in patients suffering from thrombogenic strokes, are critical in addressing and reducing adverse neurologic short- and long-term morbidities. Patient, patient’s family, nurse, and provider education of early neurologic compromise signs or symptoms to look for in patients with COVID-19 infection should be discussed and understood. It’s important to recognize that data from the DEFUSE 3 trial has increased the potential window for mechanical intervention in stroke patients up to 16 hours [18]. This window expansion provides a greater potential possibility for close neurologic examination at the local patient and initial provider level to result in difference-making intervention. 

As COVID-19 disease burden slows, the ability to isolate positive COVID-19 infected patients from non-positive patients will become a significant safety and treatment factor for many medical centers throughout the globe as pandemic spread occurs. Not only is this true at the local hospital level but also within systems or geographical levels where patient transfers and movement are necessary. Disaster management algorithms should look to include not just units within the medical center level but also coordinated throughout hospital systems at a local, city, or even state level if at all possible as resources become strained or pinched in certain regions. For various hospital systems, this type of integration is openly challenged by the realities of competing hospital’s payor-mix considerations. However, with the growing onslaught of hospital consolidation in the market, this has the potential to allow for greater coordination of resources [19].

**Figure 3:**
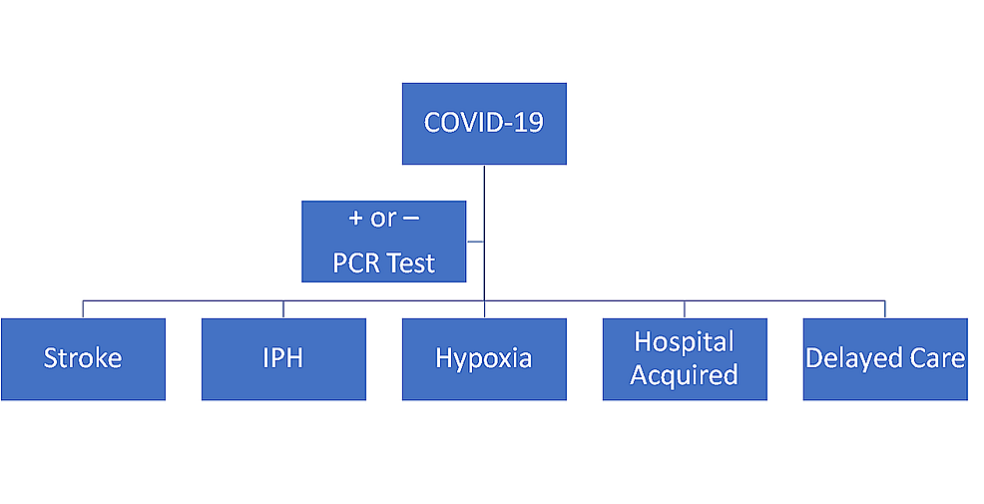
Neurological Manifestations of COVID-19 and Potential Solutions IPH: intracerebral hemorrhage, PCR: polymerase chain reaction

The most important and immediate work can be done on patient education. Patients need to be educated and informed about seeking proper care for medical conditions despite any justified fears they may have from threat of COVID-19 infection. This should be direct education to the patient through standard media techniques as well as primary care provider to patient education. Such educational endeavors certainly have the potential to improve neurological outcomes in patients delaying emergency medical evaluations and treatment. Educating patients on the importance of identifying major medical threats to their health and the need to seek medical care is the most direct and impactful change that could dictate care at the local and community levels. Educating patients, systems of providers, and ancillary healthcare staff about how to best communicate with patients is essential to inform them of urgent treatment needs and where care can be rendered, relieve concerns that are heightened during trying times such as the current pandemic, and aid in understanding or empowering individuals to be active in their health and medical care needs. This situation obviously extends well beyond neurological issues and can impact oncologic care, pediatric vaccinations, preventative medicine, cardiac care, and essentially all other medical conditions which had been impacted by concerns that COVID-19 had throughout various health care markets and regions. Neurological manifestations and potential solutions are visually displayed in Figure 3. 

## Conclusions

The COVID-19 pandemic affects not only the respiratory status of those infected, but also can contribute to end-organ dysfunction and neurologic compromise. Knowledge of such neurologic manifestations must be assessed and addressed in the initial stage and throughout the care of COVID-19-infected patients in order to prevent and reduce adverse neurologic injury when encountered. Familiarity and integration of the latest stroke intervention criteria combined with the best possible neurologic examination are critical given the many manifestations that this virus has on the human body. Further investigation is warranted to improve the understanding of this disease at a pathophysiologic, infectious disease, radiographic, and clinical as well as basic science level. As the military medical systems are called into action, we must quickly incorporate fast-changing disease treatment paradigms while applying the scientific process to identify best practices and lessons learned to apply in further missions to combat the pandemic. Open communication and supportive educational endeavors on critical care training are essential to ensure that our military medical teams are effectively able to address this disease when called upon during current and future crises related to COVID-19.
